# Cardiomyopathy Related to a Weight Loss Supplement: A Case Report and Review of Literature

**DOI:** 10.1177/2324709617711462

**Published:** 2017-06-02

**Authors:** Ghulam Murtaza, Sujeen Adhikari, Irfan Siddiqui, Hannah Lu, Aneesh Kuruvilla

**Affiliations:** 1 Advocate Christ Medical Center, Oak Lawn, IL, USA; 2 Rosalind Franklin University of Medicine and Science, North Chicago, IL, USA

**Keywords:** cardiomyopathy, heart failure, xenadrine, herbal supplement

## Abstract

There are various etiologies of dilated cardiomyopathy. However, in young patients without a strong family history of cardiovascular disease, alcohol or drug abuse, viral infections, and absence of endocrine and metabolic abnormalities, ischemia is an unlikely cause. We present an interesting case of a young female without traditional risk factors who developed dilated cardiomyopathy following administration of a weight loss supplement xenadrine and had resolution of symptoms after discontinuation of the supplement.

## Introduction

Of the many causes of dilated cardiomyopathy, ischemia is the most common cause. However, in younger patients without risk factors, ischemia is an unlikely cause. Instead, viruses, drugs, medications, and autoimmune and metabolic abnormalities are the usual culprits of dilated cardiomyopathy in the younger population. At times when the etiology of dilated cardiomyopathy cannot be determined, it is termed as idiopathic dilated cardiomyopathy, which has been shown to present at younger age than other forms of cardiomyopathies.^1^ Xenadrine is a weight loss supplement used mainly because it increases the basal metabolic rate.^2,3^ We present a rare case of xenadrine-induced cardiomyopathy in a young female.

## Case Presentation

We present the case of a 28-year-old female with a past medical history of bronchial asthma who was transferred from an outside hospital for further management of systolic heart failure. The patient presented with chest discomfort and laboratory results showed an elevated beta-natriuretic peptide level and elevated troponin of 54.5. Heart rate on admission was 99 beats per minute, blood pressure was 118/69 mm Hg, and saturation was 99% on room air.

Electrocardiogram showed sinus tachycardia with nonspecific ST changes in leads II, III, and AVF. Transthoracic echocardiogram revealed an ejection fraction (EF) of 30% to 35% and hypokinesia of the inferior wall. She subsequently underwent a cardiac catheterization, which revealed clean coronary arteries with an EF of 20% to 25% and moderate diffuse hypokinesis of the left ventricular wall. She did not have symptoms suggesting a viral etiology. She did not have a family history of coronary artery disease or sudden cardiac death. Her past 2 pregnancies were successful with no cardiac complications. Further history revealed that she had been going through a difficult time and recently started using supplements to help her lose weight. She started using xenadrine weight loss supplement 1 month ago and took 8 tablets per day. Prior to starting weight loss supplements, the patient was ambulating without any difficulty. She denied paroxysmal nocturnal dyspnea or orthopnea. She could climb a few flight of stairs with ease. She denied tobacco, marijuana, cocaine, heroin, or LSD use. At the hospital, the patient was advised to discontinue the weight loss supplement.

On presentation to our hospital, her urine toxicology screen was negative. A complete respiratory panel was negative, and an autoimmune panel including anti-nuclear antibody and anti-dsDNA was negative. Her beta-natriuretic peptide was elevated to 572 pg/mL on presentation. Her creatinine and sodium and other electrolytes were unremarkable. Liver function tests were normal. Complete blood count was normal as was lactic acid. Vital signs were stable. Chest X-ray showed some vascular congestion.

On physical examination, the patient was lying comfortably in no acute distress. She had mild pitting edema in the lower extremities. No jugular venous distention was noted. Auscultation of the heart revealed sinus rhythm with normal S1 and S2. Basilar crackles were heard on auscultation of the lungs. Right heart catheterization was done, which showed cardiac index of 3.44 L/min/m^2^, pulmonary capillary wedge pressure of 25 mm Hg, central venous pressure of 15 mm Hg, and mean pulmonary artery pressure of 42 mm Hg.

The patient was started on aspirin, furosemide, spironolactone, enalapril, and metoprolol. Follow-up with 2-dimensional echocardiography 1 week later showed an EF of 45%. The patient was discharged home in stable condition with instructions for outpatient follow-up. She was recently seen in clinic and was doing well.

## Discussion

Our patient was 28 years old without any risk factors for cardiomyopathy. She denied tobacco and alcohol use. She denied a family history of cardiac disease and denied upper respiratory symptoms in the preceding weeks to suggest a viral etiology. Genetic testing was not considered. Lastly, she did not have rheumatic heart disease as a child. In the absence of any identifiable risk factors for dilated cardiomyopathy and the fact that her ejection fraction and symptoms improved after discontinuation of xenadrine, the etiology for the dilated cardiomyopathy was likely related to the patient’s use of xenadrine supplements.

Herbal products have become readily available and their use has been increasing. Products containing ephedra accounted for more than 60% of all adverse reactions to herbs in the United States, yet these products accounted for less than 1% of herbal product sales.^4^ Xenadrine RFA includes ephedra alkaloid and caffeine.^5^ Ephedra, also known as ma huang, was commonly used in the past for weight loss. Although the exact mechanism is not known, it is believed that it causes direct myocyte injury due to elevated catecholamine levels, which decrease the viability of cardiac myocytes.^6^ The free radicals from the catecholamine disrupt sodium and calcium transporters, which contributes to myocardial dysfunction. Another proposed mechanism is that the excessive catecholamine release induces myocardial injury by causing vascular spasm, which can lead to myocarditis and/or myocardial infarction.^7,8^ Ephedra may be associated with systolic dysfunction, arrhythmias, and death.^9,10^ Other side effects include elevated blood pressure and potassium and glucose abnormalities.^5^

There have been case studies and case reports on ephedra’s effect on the heart. Withdrawal of this agent, in conjunction with proven pharmacotherapy, results in significant improvement in functional status and left ventricular EF.^10^ Peters et al showed that with use of ephedra for as less as a month to 2 years, EF dropped an average of 20 ± 5%.^9^ Our case was similar as our patient used the supplement for 30 days, and her EF dropped to 20% to 25%. All the patients were treated with regular angiotensin-converting enzyme inhibitors and beta-blockers in the study by Peters et al, as in our case. The EF normalized within a median of 6 months for all 6 patients including our patient.^9^

## Conclusion

In young patients without any risk factors who present with signs and symptoms of new-onset heart failure, a thorough history should be obtained and suspicion for weight loss and energy supplements should be high on the differential diagnosis. Conventional heart failure therapy and cessation of ephedra has shown to reverse the decreased ejection fraction and improve symptoms.
